# Association of SCARB1 Polymorphisms with Coronary Artery Disease Risk: A Systematic Review and Meta-Analysis

**DOI:** 10.3390/genes17070768

**Published:** 2026-06-30

**Authors:** Jinzhou Yu, Qianyu Zhou, Qiang Zhang, Jifeng Sun, Mengting Liu, Mingyang Zhao, Tong Wanyan, Yulong Wan, Changqing Sun, Hua Ye, Lianke Wang

**Affiliations:** 1 School of Nursing and Health, Zhengzhou University, Zhengzhou 450001, China; yjz1997@connect.hku.hk (J.Y.); suncq@zzu.edu.cn (C.S.); 2 College of Public Health, Zhengzhou University, Zhengzhou 450001, China; 3 Biological Sciences, De Anza College, Cupertino, CA 95014, USA; 4 State Key Laboratory of Metabolic Dysregulation & Prevention and Treatment of Esophageal Cancer, Zhengzhou University, Zhengzhou 450001, China; 5 Henan Key Laboratory of Tumor Epidemiology and Henan International Joint Laboratory of Tumor Biomarkers and Molecular Imaging, Zhengzhou 450001, China

**Keywords:** SCARB1, genetic polymorphism, coronary artery disease, meta-analysis, genetic susceptibility

## Abstract

Background: Coronary artery disease (CAD) remains a major cause of morbidity and mortality worldwide. Variants in the lipid metabolism gene SCARB1 may influence CAD susceptibility, but existing evidence is inconsistent. Methods: We systematically searched PubMed, Embase, Web of Science, the Cochrane Library, and Scopus up to 13 February 2026 for case–control studies on SCARB1 polymorphisms and CAD risk. Three polymorphisms, rs5888, rs4238001, and rs10846744, were included. Pooled odds ratios (ORs) and 95% confidence intervals (CIs) were calculated under multiple genetic models. Subgroup analyses were conducted for rs5888 by sex, ethnicity, and clinical outcome. Heterogeneity was assessed using I^2^. Results: 12 eligible case–control studies involving 3947 CAD cases and 5076 controls were included. Overall, rs5888 was not significantly associated with CAD in any genetic model, either in the pooled analysis or in subgroup analyses by ethnicity and clinical outcome. In sex-stratified analyses, males carrying the TT genotype had a significantly lower CAD risk under the recessive model (OR = 0.73, 95% CI: 0.57–0.93), whereas no significant association was observed in females. No significant association was found between rs4238001 and CAD under any model. In contrast, the rs10846744 G allele was significantly associated with reduced CAD risk under the allelic (OR = 0.78, 95% CI: 0.64–0.94), dominant (OR = 0.68, 95% CI: 0.50–0.93), homozygote (OR = 0.65, 95% CI: 0.44–0.94), and additive models (OR = 0.80, 95% CI: 0.67–0.96). Conclusions: SCARB1 rs5888 showed a male-specific association with CAD, while rs10846744 showed a suggestive inverse association that requires further validation.

## 1. Introduction

Coronary artery disease (CAD) remains a leading cause of mortality and long-term disability worldwide, and its burden continues to challenge health systems and societal productivity [1]. CAD is driven by a multilevel risk architecture; modifiable exposures such as smoking, diet, physical inactivity, and metabolic dysfunction interact with inherited susceptibility that shapes lipid handling, vascular inflammation, and atherosclerotic progression [2]. In contemporary cardiovascular prevention, risk assessment is increasingly moving from traditional clinical models toward integrated frameworks that combine phenotypic and genetic information [3,4]. In this context, interrogating single-nucleotide polymorphisms (SNPs) associated with CAD is important for improving etiologic insight into atherosclerosis-related pathways and for informing future mechanistic and translational research [5,6].

The SCARB1 gene is located on chromosome 12 and encodes scavenger receptor class B type 1 (SR-BI), an HDL receptor involved in lipid transport and cholesterol exchange [7]. SR-BI plays a central role in lipoprotein metabolism and reverse cholesterol transport by mediating selective hepatic uptake of HDL-derived cholesterol, thereby contributing to systemic cholesterol homeostasis and atherosclerosis-related pathways [7]. Multiple common SNPs have been reported in SCARB1, including rs5888, rs10846744, and rs4238001, which have been investigated in human genetic association studies [8,9]. Genetic variation in SCARB1 has been implicated in lipid metabolism, and its encoded receptor SR-BI is increasingly recognized as a potential therapeutic target in atherosclerosis, including CAD [10].

However, evidence from individual association studies on SCARB1 polymorphisms and CAD risk has remained inconsistent. For rs5888, case–control studies in different populations have reported heterogeneous findings, including protective associations, null results, and directionally discordant effects [8,11,12,13,14,15,16,17,18,19]. For rs10846744, some studies suggested that the G allele was associated with lower CAD risk, but limited sample size and population heterogeneity constrained generalizability [10,12,19]. For rs4238001, which has also attracted attention because of potential functional relevance, available reports similarly failed to converge on a stable risk pattern [12,14,20]. These discrepancies likely reflect differences in case definition, ethnic background, study power, and analytic strategy, as well as underexplored effect modification by sex. Previous meta-analytic evidence on the association between *SCARB1* and CAD focused mainly on rs5888 [21]. However, because the number of eligible studies for rs5888 was limited, the pooled conclusion remained preliminary and required further confirmation. In addition, other common *SCARB1* polymorphisms, particularly rs10846744 and rs4238001, were not included [21].

Therefore, the present systematic review and meta-analysis synthesized genotype-based case–control evidence on the associations between three common SCARB1 polymorphisms and CAD risk, with the aim of clarifying whether the available candidate gene studies show consistent model-specific association patterns and of providing complementary evidence for future mechanism-oriented research.

## 2. Materials and Methods

This systematic review and meta-analysis were conducted in accordance with the Preferred Reporting Items for Systematic Reviews and Meta-Analyses (PRISMA) guidelines [22]. The protocol of the present study was registered at PROSPERO (ID: CRD420261307395).

### 2.1. Literature Search Strategy

A comprehensive literature search was performed in PubMed, Embase, Web of Science, Cochrane Library, and Scopus databases from inception to 13 February 2026. The search strategy combined terms related to CAD, genetic polymorphisms, and the SCARB1 gene. Medical Subject Headings (MeSH) and free-text terms were used, including CAD, coronary heart disease (CHD), myocardial infarction (MI), acute coronary syndrome (ACS), polymorphism, SNPs, SCARB1, and SR-BI. The detailed search strategies for each database are provided in Appendix A Table A1.

### 2.2. Selection Criteria

Studies were considered eligible if they met the following criteria: they were human case–control studies that examined the association between SCARB1 polymorphisms (rs5888, rs4238001, and/or rs10846744) and the risk of CAD. CAD was defined to include CAD, CHD, MI, or premature CAD (PCAD). Eligible studies were required to report sufficient genotype or allele frequency data in both cases and controls to allow calculation of odds ratios with corresponding 95% confidence intervals. Only studies published in English were included.

Studies were excluded if they involved special populations that were not comparable with the general population, such as extreme pedigrees or patients with major comorbid conditions that could substantially confound the association. Studies were also excluded if they were genome-wide association studies, focused on rare SCARB1 variants, lacked an appropriate control group, or did not report extractable genetic data.

### 2.3. Data Extraction

Two investigators (JZY and QYZ) independently extracted data from each eligible study using a standardized data extraction form. The extracted information included the first author and year of publication, study population country and ethnicity, CAD phenotype, sample size, mean age and sex distribution in cases and controls, the SCARB1 polymorphisms examined, and genotype and allele frequency data for cases and controls. The Hardy–Weinberg equilibrium (HWE) status of the control group was also recorded and was calculated when unavailable. Any discrepancies in data extraction were resolved through discussion to ensure accuracy and consistency of the extracted data.

### 2.4. Quality Assessment

The methodological quality of the eligible case–control studies was evaluated independently by two reviewers (JZY and QYZ) according to the Newcastle-Ottawa Scale (NOS) [23]. The NOS assesses three major aspects of study design: participant selection, comparability between groups, and exposure assessment. Each study could receive up to nine stars. Studies with a total NOS score of seven stars or higher were considered to be of high methodological quality. Any disagreements in quality assessment were resolved through discussion of the specific NOS items with differing scores until consensus was reached.

### 2.5. Statistical Analysis

Statistical analyses were performed to evaluate the association between SCARB1 polymorphisms and the risk of CAD. Pooled odds ratios with corresponding 95% confidence intervals (CI) were calculated under six genetic models, including allelic, dominant, recessive, homozygote, heterozygote, and additive models. To further identify the most likely inheritance pattern for each polymorphism, the Thakkinstian algorithm was applied based on pooled comparisons among homozygous variant, heterozygous, and wild-type genotypes [24]. Between-study heterogeneity was assessed using Cochran’s Q test and quantified by the I^2^ statistic. When substantial heterogeneity was detected, a random-effects model based on the restricted maximum likelihood method was applied when I^2^ > 50%. Otherwise, a fixed-effects model was used [25]. To account for multiple comparisons, *p* values across the six genetic models were adjusted using the Benjamini–Hochberg false discovery rate (FDR) method for each SNP separately. A Max test was additionally performed to assess the overall association of each SNP with CAD while accounting for the correlation among different genetic models.

Subgroup analyses were conducted according to sex, ethnicity (Caucasians and Han Chinese), and CAD subtype to explore potential sources of heterogeneity and to assess effect modification. Ethnicity was classified into Caucasians (encompassing European, Middle Eastern, and North African groups with shared West Eurasian ancestry), and East Asians (Han Chinese), based on inferred genetic clustering and population structure [26]. Sensitivity analyses were performed by sequentially omitting individual studies to examine the stability and robustness of the pooled estimates. Additionally, a sensitivity analysis was conducted by excluding studies that deviated from HWE (*p* < 0.05) to assess the reliability of the results. Publication bias was evaluated using visual inspection of funnel plots and Egger’s regression test. All statistical tests were two-sided, and a *p* value < 0.05 was considered statistically significant. All analyses were conducted using Stata/MP 18.0 for macOS (StataCorp LLC, College Station, TX, USA).

## 3. Results

### 3.1. Search Results, Eligible Study Characteristics, and Quality Assessment

**Figure 1:**
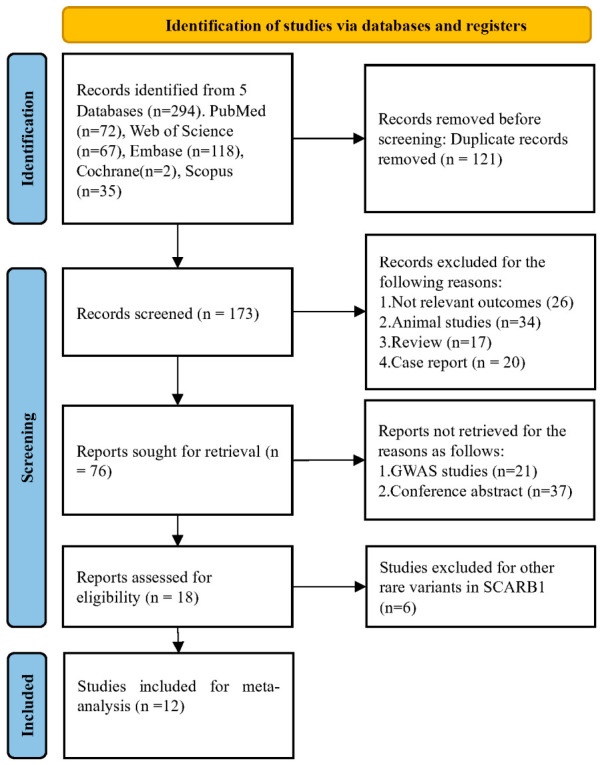
Flowchart of the study selection process.

The literature search identified a total of 294 records through database searching. After removal of duplicates (*n* = 121) and review and animal studies, 76 potentially relevant articles were retrieved for full-text evaluation. Following application of the inclusion and exclusion criteria, 12 eligible case–control studies were finally included in this meta-analysis [8,10,11,12,13,14,15,16,17,18,19,20,27]. The study selection process is illustrated in Figure 1. Table 1 shows the main characteristics of the included studies. The included sample sizes were 3038 cases and 4926 controls for rs5888, 447 cases and 459 controls for rs10846744, and 462 cases and 343 controls for rs4238001. The study populations covered multiple ethnic groups, including Asian, Middle Eastern, African, and European populations, and the outcomes included CAD, CHD, MI, and PCAD. Four study datasets showed deviation from HWE in the control group, including three for rs5888 and one for rs4238001. Specifically, deviations were observed in the studies by Goodarzynejad et al., ArulJothi et al., and Saad et al. for rs5888, and by Caykara et al. for rs4238001. Table A2 shows that all included studies showed moderate to high methodological quality based on the NOS, with total scores ranging from 5 to 8.

### 3.2. The rs5888 Polymorphism

**Figure 2:**
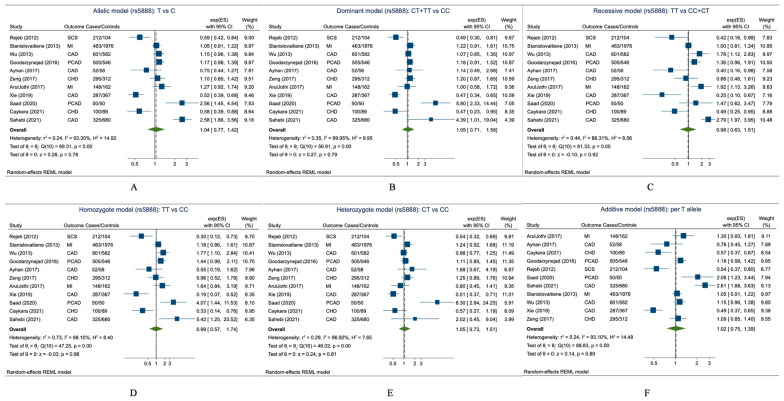
Forest plot for the association of SCARB1 rs5888 (T/C) polymorphism with coronary artery diseases risk. (**A**) allelic model, (**B**) dominant model, (**C**) recessive model, (**D**) homozygote model, (**E**) heterozygote model, (**F**) additive model.

**Figure 3:**
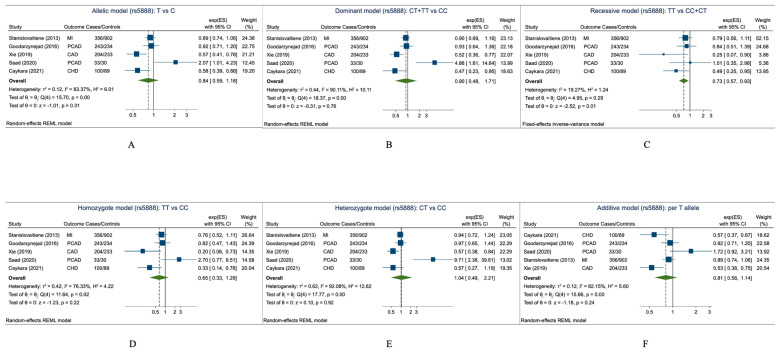
Forest plot for the association between SCARB1 rs5888 (T/C) polymorphism and coronary artery diseases risk in male. (**A**) allelic model, (**B**) dominant model, (**C**) recessive model, (**D**) homozygote model, (**E**) heterozygote model, (**F**) additive model.

**Figure 4:**
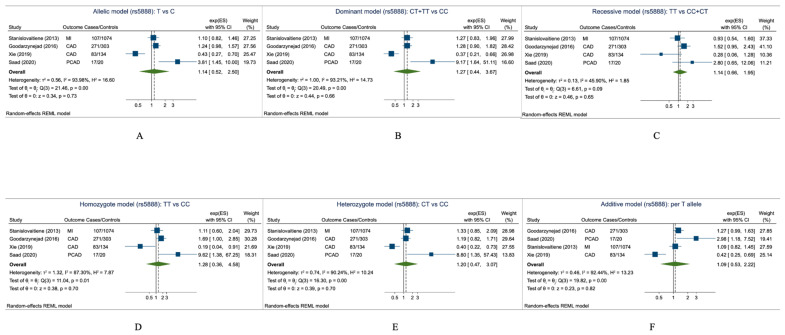
Forest plot for the association of SCARB1 rs5888 (T/C) polymorphism with coronary artery diseases risk in female. (**A**) allelic model, (**B**) dominant model, (**C**) recessive model, (**D**) homozygote model, (**E**) heterozygote model, (**F**) additive model.

**Figure A1:**
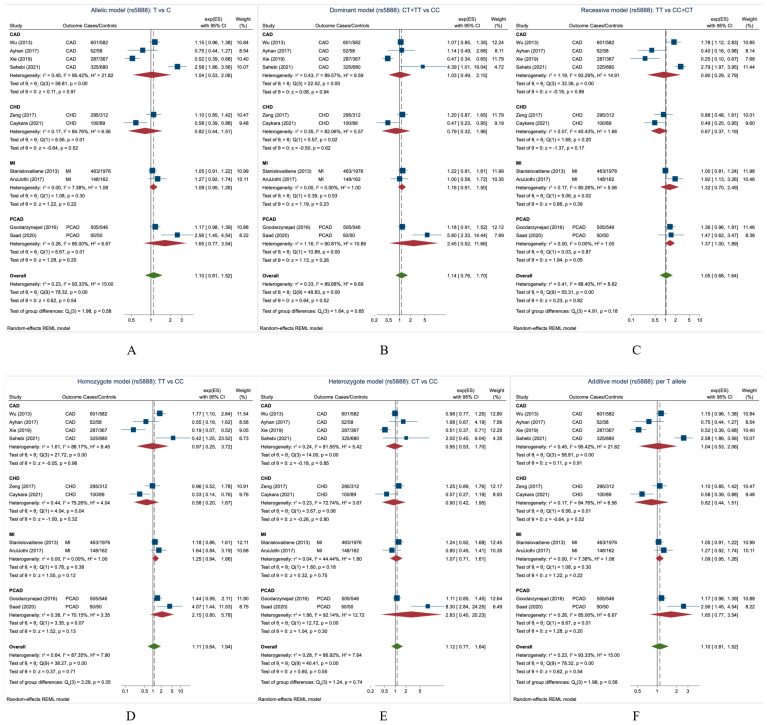
Forest plot for the association between the SCARB1 rs5888 (T/C) polymorphism and coronary artery disease risk by clinical subtype. (**A**) allelic model, (**B**) dominant model, (**C**) recessive model, (**D**) homozygote model, (**E**) heterozygote model, (**F**) additive model.

**Figure A2:**
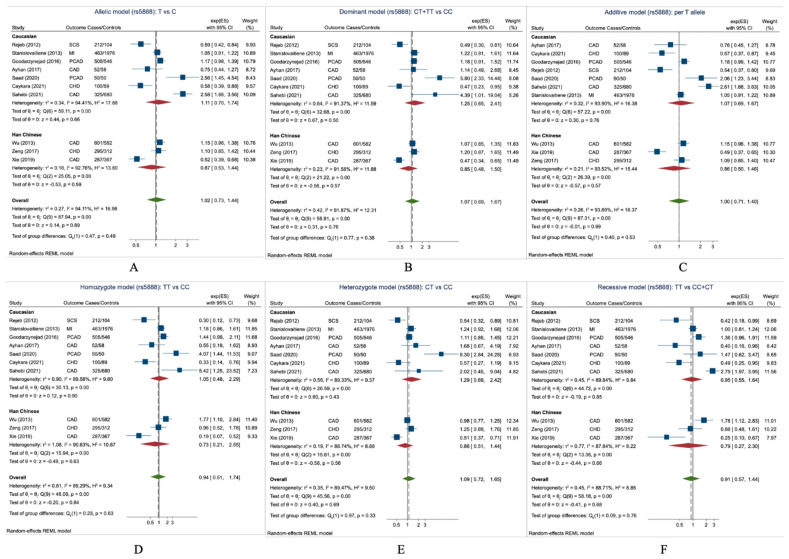
Forest plot for the association of SCARB1 rs5888 (T/C) polymorphism with coronary artery diseases risk by ethnicity. (**A**) allelic model, (**B**) dominant model, (**C**) recessive model, (**D**) homozygote model, (**E**) heterozygote model, (**F**) additive model.

Figure 2 shows that, in the overall analysis, no significant association was observed between the SCARB1 rs5888 polymorphism and CAD risk under any of the evaluated genetic models, including allelic, dominant, recessive, homozygote, heterozygote, and additive models. Sex-stratified analyses revealed a significant association between rs5888 and CAD risk in males under the recessive model. Figure 3 shows that male carriers of the TT genotype had a lower risk of CAD than carriers of the CC + CT genotypes under the recessive model (OR = 0.73, 95% CI: 0.57–0.93, *p* = 0.01). By contrast, no significant association was observed in females under any genetic model (Figure 4), and the genotype-by-sex interaction was significant (Appendix A Table A3, *p* for interaction < 0.05). Figure A1 and Figure A2 show subgroup analyses stratified by ethnicity and clinical outcome, and they revealed no statistically significant association between rs5888 and CAD risk across the six genetic models.

### 3.3. The rs4238001 Polymorphism

**Figure 5:**
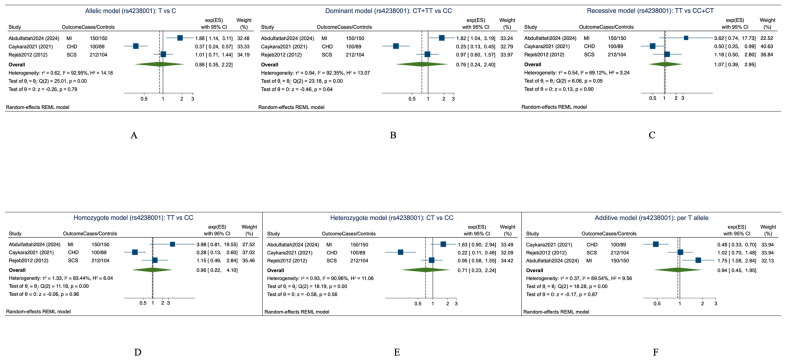
Forest plot for the association of SCARB1 rs4238001 (T/C) polymorphism with coronary artery diseases risk. (**A**) allelic model, (**B**) dominant model, (**C**) recessive model, (**D**) homozygote model, (**E**) heterozygote model, (**F**) additive model.

Figure 5 shows that there was no significant association between rs4238001 and CAD in the overall analysis across all genetic models.

### 3.4. The rs10846744 Polymorphisms

**Figure 6:**
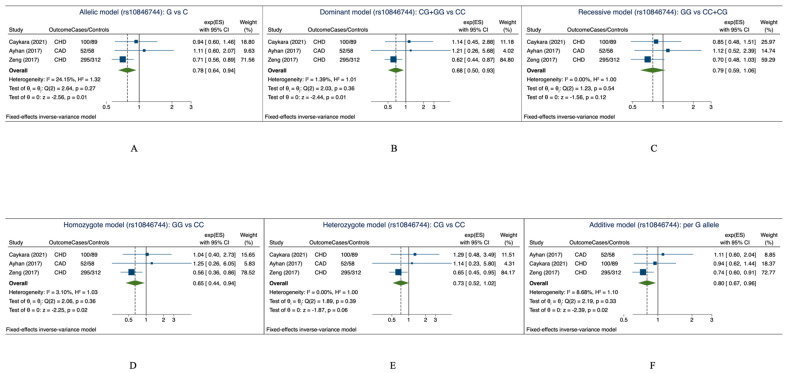
Forest plot for the association of SCARB1 rs10846744 (G/C) polymorphism with coronary artery diseases risk. (**A**) allelic model, (**B**) dominant model, (C) recessive model, (**D**) homozygote model, (**E**) heterozygote model, (**F**) additive model.

Figure 6 shows that significant associations with CAD risk were observed in the four gene models. The G allele was associated with a reduced risk of CAD under the allelic model (OR = 0.78, 95% CI: 0.64–0.94, *p* = 0.01). Similarly, significant inverse associations were identified under the dominant model (OR = 0.68, 95% CI: 0.50–0.93, *p* = 0.01), homozygote model (OR = 0.65, 95% CI: 0.44–0.94, *p* = 0.02), and additive model (OR = 0.80, 95% CI: 0.67–0.96, *p* = 0.02). No significant association was observed under the recessive or heterozygote models. Based on the pattern of pooled ORs, the association for rs10846744 appeared to be more compatible with a codominant/additive mode of inheritance rather than a purely recessive model.

Appendix A Table A4 shows that after FDR correction across the six genetic models, no significant association remained for rs5888 or rs4238001. The associations for rs10846744 in the allelic, dominant, homozygote, and additive models remained significant. Appendix A Table A5 shows that the Max test identified significant overall associations for rs5888 in the overall analysis (empirical Max test *p* = 0.016) and in males (empirical Max test *p* = 0.007), as well as for rs10846744 in the overall analysis (empirical Max test *p* = 0.034), whereas no significant association was detected for rs5888 in females (empirical Max test *p* = 0.262) or for rs4238001 (empirical Max test *p* = 0.231).

### 3.5. Heterogeneity, Sensitivity Analysis, and Publication Bias

**Figure 7:**
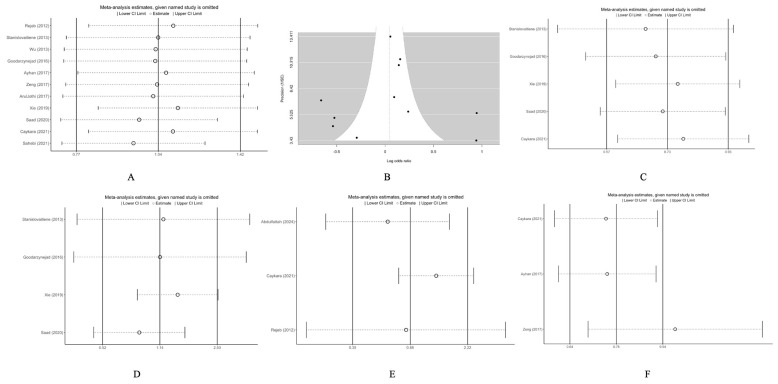
Leave-one-out sensitivity analyses and publication-bias assessment. (**A**) Leave-one-out analysis for the SCARB1 rs5888 under the allelic model. (**B**) Funnel plot for SCARB1 rs5888 under the allelic model. (**C**) Leave-one-out analysis for SCARB1 rs5888 in males under the recessive model. (**D**) Leave-one-out analysis for SCARB1 rs5888 in females under the recessive model. (**E**) Leave-one-out analysis for rs4238001 under the allelic model. (**F**) Leave-one-out analysis for SCARB1 rs10846744 under the allelic model.

**Figure A3:**
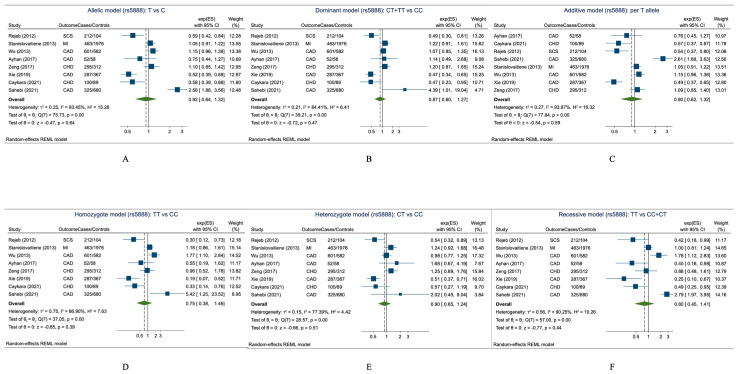
Forest plot for the association of SCARB1 rs5888 (T/C) polymorphism with coronary artery diseases risk after excluding studies that deviated from HWE. (**A**) allelic model, (**B**) dominant model, (**C**) recessive model, (**D**) homozygote model, (**E**) heterozygote model, (**F**) additive model.

**Figure A4:**
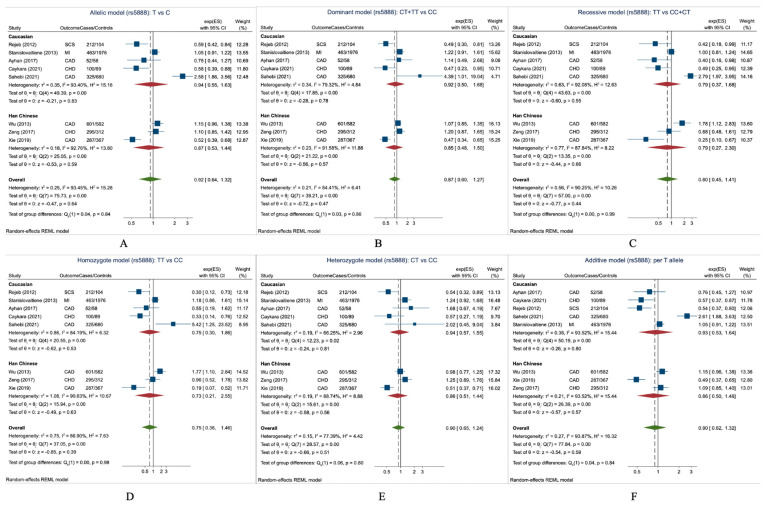
Forest plot for the association of SCARB1 rs5888 (T/C) polymorphism with coronary artery diseases risk by ethnicity after excluding studies that deviated from HWE. (**A**) allelic model, (**B**) dominant model, (**C**) recessive model, (**D**) homozygote model, (**E**) heterozygote model, (**F**) additive model.

**Figure A5:**
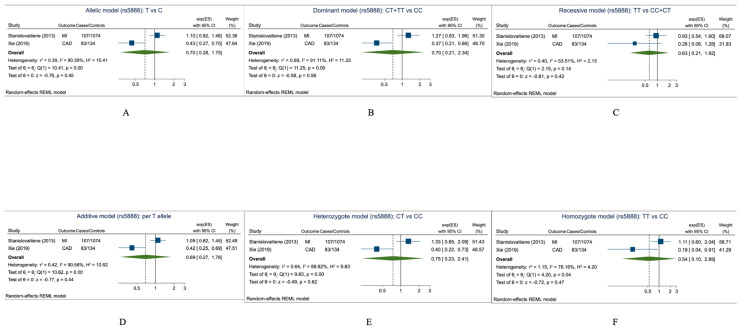
Forest plot for the association of SCARB1 rs5888 (T/C) polymorphism with coronary artery diseases risk in female after excluding studies that deviated from HWE. (**A**) allelic model, (**B**) dominant model, (**C**) recessive model, (**D**) homozygote model, (**E**) heterozygote model, (**F**) additive model.

**Figure A6:**
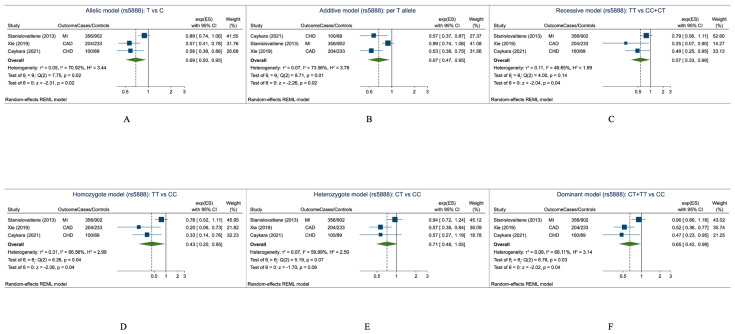
Forest plot for the association of SCARB1 rs5888 (T/C) polymorphism with coronary artery diseases risk in male after excluding studies that deviated from HWE. (**A**) allelic model, (**B**) dominant model, (**C**) recessive model, (**D**) homozygote model, (**E**) heterozygote model, (**F**) additive model.

**Figure A7:**
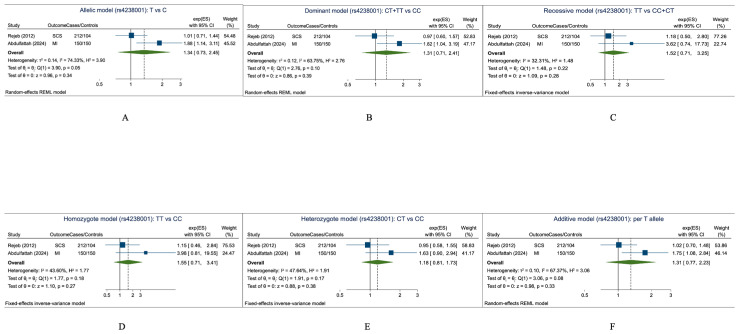
Forest plot for the association of SCARB1 rs4238001 (T/C) polymorphism with coronary artery disease risk in males after excluding studies that deviated from HWE. (**A**) allelic model, (**B**) dominant model, (**C**) recessive model, (**D**) homozygote model, (**E**) heterozygote model, (**F**) additive model.

Figure 7A,C–F show the leave-one-out sensitivity analyses under the allelic model for rs5888, rs4238001, and rs10846744, performed by sequentially omitting each individual study. The pooled estimates for rs5888 and rs4238001 remained consistent. In contrast, the leave-one-out plot for rs10846744 showed that omitting Zeng et al. substantially altered the pooled effect size and rendered the association non-significant, whereas omitting Caykara et al. or Ayhan et al. had minimal impact on the pooled estimate. Figure 7B shows no evidence of publication bias in rs5888-related studies, with no marked funnel plot asymmetry and a non-significant Egger’s test (*p* = 0.778). Publication bias was evaluated for rs5888 only, as too few studies were available for rs4238001 and rs10846744 to support funnel plots or Egger’s test. Figure A3, Figure A4, Figure A5, Figure A6 and Figure A7 show that, after excluding studies deviating from HWE, the pooled estimates remained largely unchanged, with the null associations for rs5888 overall, female-specific rs5888, ethnicity-stratified rs5888, and rs4238001 preserved, and the suggestive inverse association for rs5888 in males remaining significant.

## 4. Discussion

This systematic review and meta-analysis integrated evidence from 12 case–control studies to evaluate the association between three common SCARB1 polymorphisms, rs5888, rs4238001, and rs10846744, with CAD risk. In all populations, rs5888 showed no significant association with CAD in all genetic models, while sex-stratified analysis identified a significant protective effect of the TT genotype in males under the recessive model. rs10846744 showed a suggestive inverse association with CAD across multiple genetic models. Rs4238001 was not significantly associated with CAD in any model.

The sex-specific pattern for rs5888 is an important finding of this study. Compared with the previous meta-analysis, which suggested a protective direction for the T allele in males but did not reach statistical significance [21], our analysis provides statistically significant evidence that the TT genotype is associated with a reduced risk of CAD in males under the recessive model. This sex-specific pattern is supported by prior evidence linking SCARB1 variants to lipid phenotypes. A previous meta-analysis of 12 studies reported that the SCARB1 rs5888 variant was associated with a more favorable fasting lipid profile predominantly in non-Asian men, with higher HDL-C and lower triglyceride levels, whereas no significant associations were observed in women [28]. The precise functional consequence of the rs5888 TT genotype remains uncertain. This observed sex-specific association may be modified by sex-related hormonal factors, including oestrogen-related regulation of SR-BI expression and lipid metabolism [29,30]. In addition, the recessive model identified here should be interpreted as the statistical pattern best fitting the pooled data, rather than the only possible biological explanation.

A suggestive inverse association with the rs10846744 G allele was observed in the allelic, dominant, homozygote, and additive models, indicating a broadly consistent pattern across genetic models and possible compatibility with an additive mode of inheritance. Rs10846744 is an intronic non-coding variant in the SCARB1 region and is not located at a canonical splice site. Rather, it may mark regulatory variation within this locus [31]. Previous studies have shown that this locus is pleiotropically associated with several sterol-related and atherosclerosis-related phenotypes, and may influence SCARB1-region regulation or linked pathways relevant to SR-BI function, cholesterol flux, and atherosclerotic progression [32,33]. Although the leave-one-out analysis indicated sensitivity to exclusion of the study by Zeng et al., rs10846744 has also been supported as a CAD-related locus in genome-wide association studies [9,34].

Rs4238001 did not show a significant effect in any genetic model. This null result is informative because gene-level biological plausibility does not guarantee measurable risk effects for every common variant within the same gene [35]. Variants can differ in functional relevance, penetrance, and context dependence [36]. However, the null finding for rs4238001 should be interpreted cautiously because of the limited number of studies and participants.

In addition to the candidate gene evidence synthesized here, the broader genetic context of the SCARB1 locus should also be considered. Prior large-scale studies have linked SCARB1-region variation to subclinical atherosclerosis, incident cardiovascular disease, and CAD-related phenotypes, particularly for rs10846744 and correlated variants within the same locus [9]. In the Multi-Ethnic Study of Atherosclerosis, rs10846744 was associated with subclinical atherosclerosis and incident cardiovascular disease, and subsequent consortium-level analyses further supported its association with CAD events [9,34]. This evidence strengthens the biological and epidemiological plausibility of our finding for rs10846744 and further indicates that the effects of individual SNPs should be interpreted in the context of regional linkage disequilibrium rather than as isolated single-variant signals. By contrast, for rs5888 and rs4238001, currently available large-scale locus-level evidence remains more limited or less consistent, which may partly explain the heterogeneous or null associations observed in candidate gene studies [13,27].

Our findings suggest that the genetic effects of SCARB1 may be variant-specific and modified by sex. Future studies should therefore prespecify sex-stratified analyses, formally test genotype-by-sex interactions, and use standardized CAD definitions. Further functional studies are needed to clarify the biological mechanisms underlying these associations, particularly their relevance to cholesterol transport and CAD susceptibility.

There are several limitations to this meta-analysis. First, heterogeneity was present in selected models, which is expected given differences in ethnicity, CAD phenotype definitions, and study design. We addressed this issue through subgroup analyses and sensitivity analyses, and the main conclusions remained directionally stable. Second, the geographic distribution of the available studies was limited, with relatively few studies from Europe and other underrepresented regions, which may restrict the generalizability of the findings. Third, because fewer studies were available for rs10846744 and rs4238001, the pooled estimates should be interpreted with caution, especially for rs10846744, whose association was sensitive to an individual study in the leave-one-out analysis. Fourth, although the Newcastle-Ottawa Scale was used for general methodological appraisal, it does not specifically assess genotyping quality or other sources of bias unique to genetic association studies. Finally, all included studies were case–control studies, so causal inference is limited, and the present findings are insufficient to support their use in CAD risk prediction.

## 5. Conclusions

In conclusion, our meta-analysis suggests that SCARB1 may influence CAD risk in a sex-modified and variant-specific manner. The rs5888 TT genotype was associated with a reduced risk of CAD in males, the rs10846744 G allele showed a suggestive inverse association, whereas rs4238001 showed no significant association. Further large-scale multi-ethnic studies and functional studies are warranted to confirm the robustness and biological basis of these associations. Our findings may help clarify prior inconsistencies and provide updated evidence on the potential role of SCARB1 variation in CAD, while informing future mechanism-oriented research.

## Figures and Tables

**Table 1:** Summary of included studies.

First Author	Year	Ethnicity	Outcome	N (Case/Control)	Age (Case)	Age (Control)	SNPs	Genotype CC (Case/Control)	Genotype CT/CG (Case/Control)	Genotype TT/GG (Case/Control)	HWE
Rejeb	2012	Tunisia	SCS	212/104	60.6 ± 10.6	59.4 ± 11.9	rs5888	105/34	96/58	11/12	0.087
Stanislovaitiene	2013	Lithuania	MI	463/1976	NA	NA	rs5888	67/337	233/943	163/696	0.564
Wu	2013	Han Chinese	CAD	601/582	62.3 ±10.6	61.5 ± 10.9	rs5888	324/324	224/228	53/30	0.210
Goodarzynejad	2016	Iran	PCAD	505/546	45.6 ± 5.9	45.6 ± 6.1	rs5888	162/195	259/281	84/70	0.044
Ayhan	2017	Turkey	CAD	52/58	60.0 ± 6.8	57.1 ± 10.8	rs5888	13/16	30/22	9/20	0.070
Zeng	2017	Han Chinese	CHD	295/312	67.0 ± 11.2	67.0 ± 9.5	rs5888	160/183	114/104	21/25	0.068
ArulJothi	2017	Tamil	MI	148/162	M: 52.0 ± 1.0 F:55.0 ± 1.5	M: 51.1 ± 1.7 F: 52.2 ± 1.6	rs5888	32/35	71/97	45/30	0.011
Xie	2019	Han Chinese	CAD	287/367	M: 59.9 ± 9.2 F: 61.01 ± 8.3	M: 61.3 ± 8.9 F: 58.7 ± 8.5	rs5888	193/180	89/163	5/24	0.107
Saad	2020	Egyptian	PCAD	50/50	46.7 ± 5.7	47.4 ± 7.1	rs5888	9/28	24/9	17/13	<0.001
Caykara	2021	Turkey	CHD	100/89	57.1 ± 7.3	55.9 ± 4.7	rs5888	30/15	51/45	19/29	0.728
Sahebi	2021	Iran	CAD	325/680	61.5 ± 8.9	49.6 ± 7.6	rs5888	2/18	45/200	278/462	0.508
Caykara	2021	Turkey	CHD	100/89	57.07 ± 7.3	55.9 ± 4.7	rs10846744	10/10	40/31	50/48	0.163
Ayhan	2017	Turkey	CAD	52/58	60.0 ± 6.8	57.1 ± 10.8	rs10846744	3/4	18/21	31/33	0.793
Zeng	2017	Han Chinese	CHD	295/312	67.0 ± 11.2	66.5 ± 13.3	rs10846744	119/92	116/137	60/83	0.058
Rejeb	2012	Tunisia	SCS	212/104	60.6 ± 10.6	59.4 ± 11.9	rs4238001	85/41	108/55	19/8	0.073
Caykara	2021	Turkey	CHD	100/89	57.07 ± 7.3	55.9 ± 4.7	rs4238001	65/28	18/35	17/26	0.044
Abdulfattah	2024	Iraqi	MI	150/150	52.1 ± 9.1	50.6 ± 6.0	rs4238001	110/125	33/23	7/2	0.434

SCS: Significant Coronary Stenosis, MI: Myocardial Infarction, CAD: Coronary Artery Disease, PCAD: Premature Coronary Artery Disease, CHD: Coronary Heart Disease, HWE: Hardy–Weinberg Equilibrium, NA: Not available, M: Male, F: Female.

## Data Availability

All data analyzed in this study were extracted from previously published studies included in this article.

## Abbreviations

The following abbreviations are used in this manuscript:

LD	linear dichrois;
ACS	acute coronary syndrome;
CAD	coronary artery disease;
CHD	coronary heart disease;
CI	confidence interval;
HWE	Hardy–Weinberg equilibrium;
HDL	high-density lipoprotein;
MeSH	Medical Subject Headings;
MI	myocardial infarction;
NOS	Newcastle–Ottawa Scale;
OR	odds ratio;
PCAD	premature coronary artery disease;
PRISMA	Preferred Reporting Items for Systematic Reviews and Meta-Analyses;
SCARB1	scavenger receptor class B member 1;
SCS	significant coronary stenosis;
SNP	single-nucleotide polymorphism;
SR-BI	scavenger receptor class B type 1;

## Appendix A

**Table A1:** Search strategy in 4 databases (PubMed, Embase, Cochrane and Web of Science). (Searched on 13 February 2026).

Database	Searched Items	Number
PubMed	#1: “Coronary Disease” [Mesh] OR “Myocardial Infarction” [Mesh] OR “Acute Coronary Syndrome” [Mesh] OR “Angina Pectoris” [Mesh] OR “coronary heart disease” [Title/Abstract] OR “coronary artery disease” [Title/Abstract] OR “coronary disease” [Title/Abstract] OR “ischemic heart disease” [Title/Abstract] OR “ischaemic heart disease” [Title/Abstract] OR “acute coronary syndrome” [Title/Abstract] OR “myocardial infarction” [Title/Abstract] OR “angina pectoris” [Title/Abstract] OR angina [Title/Abstract] OR CHD [Title/Abstract] OR CAD [Title/Abstract] OR IHD [Title/Abstract] OR MI [Title/Abstract] OR ACS [Title/Abstract] OR “coronary stenosis” [Title/Abstract] OR “coronary artery stenosis” [Title/Abstract] OR “significant coronary stenosis” [Title/Abstract] OR “coronary atherosclerosis” [Title/Abstract] OR “coronary artery atherosclerosis” [Title/Abstract]	729,682
	#2: “Polymorphism, Single Nucleotide” [Mesh] OR “Polymorphism, Genetic” [Mesh] OR polymorphism* [Title/Abstract] OR “single nucleotide polymorphism” [Title/Abstract] OR SNP [Title/Abstract] OR SNPs [Title/Abstract] OR genotype* [Title/Abstract] OR variant* [Title/Abstract] OR mutation* [Title/Abstract] OR “genetic variant*” [Title/Abstract]	1,744,106
	#3: SCARB1 [Title/Abstract] OR “SR-BI” [Title/Abstract] OR SRBI [Title/Abstract] OR “scavenger receptor class B type I” [Title/Abstract] OR “scavenger receptor class B type 1” [Title/Abstract] OR “scavenger receptor class B member 1” [Title/Abstract]	2585
	#1 AND #2 AND #3	72
Embase	#1: ‘coronary artery disease’/exp OR ‘coronary heart disease’/exp OR ‘coronary disease’/exp OR ‘myocardial infarction’/exp OR ‘acute coronary syndrome’/exp OR ‘angina pectoris’/exp OR (‘coronary artery disease’ OR ‘coronary heart disease’ OR ‘coronary disease’ OR ‘ischemic heart disease’ OR ‘ischaemic heart disease’ OR ‘myocardial infarction’ OR ‘acute coronary syndrome’ OR angina OR ‘angina pectoris’ OR CHD OR CAD OR IHD OR MI OR ACS OR ‘coronary stenosis’ OR ‘coronary artery stenosis’ OR ‘significant coronary stenosis’ OR ‘coronary atherosclerosis’ OR ‘coronary artery atherosclerosis’):ti,ab,kw	1,350,656
	#2: (‘single nucleotide polymorphism’/exp OR ‘genetic polymorphism’/exp) OR (polymorphism* OR “single nucleotide polymorphism” OR SNP OR SNPs OR genotype* OR variant* OR mutation* OR “genetic variant*”):ti,ab,kw	2,437,434
	#3: (SCARB1 OR “SR-BI” OR SRBI OR “scavenger receptor class B type I” OR “scavenger receptor class B type 1” OR “scavenger receptor class B member 1”):ti,ab,kw	3408
	#1 AND #2 AND #3	118
Web of Science	#1: TI = (“coronary disease” OR “coronary heart disease” OR “coronary artery disease” OR “ischemic heart disease” OR “ischaemic heart disease” OR “acute coronary syndrome” OR “myocardial infarction” OR angina OR “angina pectoris” OR CHD OR CAD OR IHD OR MI OR ACS OR “coronary stenosis” OR “coronary artery stenosis” OR “significant coronary stenosis” OR”coronary atherosclerosis” OR “coronary artery atherosclerosis”) OR AB = (“coronary disease” OR “coronary heart disease” OR “coronary artery disease” OR “ischemic heart disease” OR “ischaemic heart disease” OR “acute coronary syndrome” OR “myocardial infarction” OR angina OR “angina pectoris” OR CHD OR CAD OR IHD OR MI OR ACS OR “coronary stenosis” OR “coronary artery stenosis” OR “significant coronary stenosis” OR “coronary atherosclerosis” OR “coronary artery atherosclerosis”)	666,402
	#2: TI = (polymorphism* OR “single nucleotide polymorphism” OR SNP OR SNPs OR genotype* OR variant* OR mutation* OR “genetic variant*”) OR AB = (polymorphism* OR “single nucleotide polymorphism” OR SNP OR SNPs OR genotype* OR variant* OR mutation* OR “genetic variant*”)	2,133,181
	#3: TI = (SCARB1 OR “SR-BI” OR SRBI OR “scavenger receptor class B type I” OR “scavenger receptor class B type 1” OR “scavenger receptor class B member 1”) OR AB = (SCARB1 OR “SR-BI” OR SRBI OR “scavenger receptor class B type I” OR “scavenger receptor class B type 1” OR “scavenger receptor class B member 1”)	3118
	#1 AND #2 AND #3	67
Cochrane library	#1: MeSH descriptor: [Coronary Disease] explode all trees	198,22
	#2: MeSH descriptor: [Myocardial Infarction] explode all trees	158,35
	#3: MeSH descriptor: [Acute Coronary Syndrome] explode all trees	3177
	#4: MeSH descriptor: [Angina Pectoris] explode all trees	5596
	#5: (coronary NEXT heart NEXT disease* OR coronary NEXT artery NEXT disease* OR coronary NEXT disease* OR ischemic NEXT heart NEXT disease* OR ischaemic NEXT heart NEXT disease* OR acute NEXT coronary NEXT syndrome* OR myocardial NEXT infarction* OR angina OR angina NEXT pectoris OR CHD OR CAD OR IHD OR MI OR ACS OR coronary NEXT stenosis OR coronary NEXT artery NEXT stenosis OR significant NEXT coronary NEXT stenosis OR coronary NEXT atherosclerosis OR coronary NEXT artery NEXT atherosclerosis):ti,ab,kw	92,515
	#6: #1 OR #2 OR #3 AND #4 OR #5	93,035
	#7: MeSH descriptor: [Polymorphism, Single Nucleotide] explode all trees	2565
	#8: (polymorphism* OR single NEXT nucleotide NEXT polymorphism* OR SNP OR SNPs OR genotype* OR variant* OR mutation* OR genetic NEXT variant*):ti,ab,kw	45,111
	#9: #7 OR #8	45,111
	#10: (SCARB1OR SR-BI OR SRBI OR scavenger NEXT receptor NEXT class NEXT B NEXT type NEXT I OR scavenger NEXT receptor NEXT class NEXT B NEXT type NEXT 1OR scavenger NEXT receptor NEXT class NEXT B NEXT member NEXT 1):ti,ab,kw	33
	#6 AND #9 AND #10	2
Scopus	#1: TITLE-ABS-KEY (“coronary disease” OR “coronary heart disease” OR “coronary artery disease” OR “ischemic heart disease” OR “ischaemic heart disease” OR “acute coronary syndrome” OR “myocardial infarction” OR angina OR “angina pectoris” ORCHD OR CAD OR IHD OR MI OR ACS OR “coronary stenosis” OR “coronary artery stenosis” OR “significant coronary stenosis” OR “coronary atherosclerosis” OR “coronary artery atherosclerosis”)	147,294
	#2: (TITLE-ABS-KEY (polymorphism OR “single nucleotide polymorphism” OR SNP OR SNPs OR genotype OR variant OR mutation OR “genetic variant”))	2,910,229
	#3: (TITLE-ABS-KEY (SCARB1 OR “SR-BI” OR SRBI OR “scavenger receptor class B type I” OR “scavenger receptor class B type 1” OR “scavenger receptor class B member 1”))	3880
	#1 AND #2 AND #3	35

* indicates a wildcard character used in the database search strategy.

**Table A2:** Assessment of quality of included studies (Newcastle-Ottawa Quality Assessment Scale for case–control study).

Study	Term 1	Term 2	Term 3	Term 4	Term 5	Term 6	Term 7	Term 8	Total
Rejeb (2012)	★	★	★	★	★★	★	★	0	8
Stanislovaitiene (2013)	★	★	★	0	★★	★	★	0	7
Wu (2013)	★	★	★	★	★★	★	★	0	8
Goodarzynejad (2016)	★	★	★	★	★★	★	★	0	8
Ayhan (2017)	★	0	0	★	0	★	★	0	5
Zeng (2017)	★	★	★	★	★	★	★	0	7
ArulJothi (2017)	★	★	★	★	0	★	★	0	6
Xie (2019)	★	★	★	★	★★	★	★	0	8
Saad (2020)	★	★	★	★	★	★	★	0	7
Caykara (2021)	★	★	★	★	★★	★	★	0	8
Sahebi (2021)	★	★	★	★	★★	★	★	0	8
Abdulfattah (2024)	★	★	★	★	★	★	★	0	7

★ indicates one star, and ★★ indicates two stars in the Newcastle–Ottawa Scale assessment. Term 1, Is the case definition adequate?; Term 2, Representativeness of the cases; Term 3, Selection of controls; Term 4, Definition of controls; Term 5, Comparability of cases and controls on the basis of the design or analysis controlled for confounders; Term 6, Ascertainment of exposure; Term 7, Same method of ascertainment for cases and controls; Term 8, Non-response rate.

**Table A3:** Sex interaction analysis for SCARB1 rs5888 under the recessive model.

SNP	Genetic Model	Male, OR (95% CI)	Female, OR (95% CI)	*p* for Interaction
rs5888	Recessive (TT vs. CC + CT)	0.73 (0.57–0.93)	1.14 (0.66–1.95)	0.019

**Table A4:** False discovery rate-corrected (FDR-corrected) *p* values for the six genetic models of each SCARB1 polymorphism.

SNP	Genetic Model	Raw P value	FDR-Adjusted *p* Value
rs5888	Allelic	0.78	0.98
rs5888	Dominant	0.79	0.98
rs5888	Recessive	0.92	0.98
rs5888	Homozygote	0.98	0.98
rs5888	Heterozygote	0.81	0.98
rs5888	Additive	0.89	0.98
rs4238001	Allelic	0.79	0.96
rs4238001	Dominant	0.64	0.96
rs4238001	Recessive	0.9	0.96
rs4238001	Homozygote	0.96	0.96
rs4238001	Heterozygote	0.56	0.96
rs4238001	Additive	0.87	0.96
rs10846744	Allelic	0.01	0.03
rs10846744	Dominant	0.01	0.03
rs10846744	Recessive	0.12	0.12
rs10846744	Homozygote	0.02	0.03
rs10846744	Heterozygote	0.06	0.072
rs10846744	Additive	0.02	0.03

**Table A5:** Maximum-test results across all correlated genetic models for the SCARB1 polymorphisms and coronary artery disease.

Analysis Group	Studies Included	Best Model by Abs Z	Best Model Z	Best Model Raw *p*	Max Abs Z	Empirical Max Test *p*
Overall rs5888	11	recessive	2.803	0.005	2.803	0.016
Male rs5888	5	additive	−3.016	0.003	3.016	0.007
Female rs5888	4	homozygote	1.556	0.120	1.556	0.262
Overall rs4238001	3	homozygote	−1.616	0.106	1.616	0.231
Overall rs10846744	3	allelic	−2.555	0.011	2.555	0.034
